# Comprehensive analysis of miRNA-mRNA regulatory pairs associated with colorectal cancer and the role in tumor immunity

**DOI:** 10.1186/s12864-023-09635-4

**Published:** 2023-11-30

**Authors:** Cheng Liu, Chun Yu, Guoxin Song, Xingchen Fan, Shuang Peng, Shiyu Zhang, Xin Zhou, Cheng zhang, Xiangnan Geng, Tongshan Wang, Wenfang Cheng, Wei Zhu

**Affiliations:** 1 Department of Gastroenterology, the First Affiliated Hospital of Nanjing Medical University, 300 Guangzhou Road, Nanjing, 210029 Jiangsu China; 2 Department of Pathology, the First Affiliated Hospital of Nanjing Medical University, Nanjing, 210029 China Jiangsu; 3 Department of Oncology, the First Affiliated Hospital of Nanjing Medical University, 300 Guangzhou Road, Nanjing, 210029 China Jiangsu; 4 Department of Science and Technology, the First Affiliated Hospital of Nanjing Medical University, Nanjing, 210029 China Jiangsu; 5 Department of Clinical Engineer, the First Affiliated Hospital of Nanjing Medical University, Nanjing, 210029 China Jiangsu

**Keywords:** miRNA, miRNA-mRNA networks, Tumor immunity, Colorectal cancer

## Abstract

**Background:**

MicroRNA (miRNA) which can act as post-transcriptional regulators of mRNAs via base-pairing with complementary sequences within mRNAs is involved in processes of the complex interaction between immune system and tumors.

In this research, we elucidated the profiles of miRNAs and target mRNAs expression and their associations with the phenotypic hallmarks of colorectal cancers (CRC) by integrating transcriptomic, immunophenotype, methylation, mutation and survival data.

**Results:**

We conducted the analysis of differential miRNA/mRNA expression profile by GEO, TCGA and GTEx databases and the correlation between miRNA and targeted mRNA by miRTarBase and TarBase. Then we detected using qRT-PCR and validated the diagnostic value of miRNA-mRNA regulator pairs by the ROC, calibration curve and DCA. Phenotypic hallmarks of regulatory pairs including tumor-infiltrating lymphocytes, tumor microenvironment, tumor mutation burden, global methylation and gene mutation were also described. The expression levels of miRNAs and target mRNAs were detected in 80 paired colon tissue samples. Ultimately, we picked up two pivotal regulatory pairs (miR-139-5p/ STC1 and miR-20a-5p/ FGL2) and verified the diagnostic value of the complex model which is the combination of 4 signatures above-mentioned in 3 testing GEO datasets and an external validation cohort.

**Conclusions:**

We found that 2 miRNAs by targeting 2 metastasis-related mRNAs were correlated with tumor-infiltrating macrophages, HRAS, and BRAF gene mutation status. Our results established the diagnostic model containing 2 miRNAs and their respective targeted mRNAs to distinguish CRCs and normal controls and displayed their complex roles in CRC pathogenesis especially tumor immunity.

**Supplementary Information:**

The online version contains supplementary material available at 10.1186/s12864-023-09635-4.

## Background

Colorectal cancer (CRC) where the incidence ranked third has a third largest estimated mortality for all types of cancers in 2023 according to the World Health Organization (WHO) [1]. According to the statistical study of John V et al., from 2004 to 2015, the proportion of persons diagnosed with CRC at an age younger than 50 years has continued to increase, and younger adults present with more advanced disease over the past decade [2].

MicroRNAs (miRNAs), families of small noncoding RNAs, had been reported that were critical for the progression of cancers by influencing proliferation, invasion and metastasis [3]. MiRNAs can regulate gene expression at posttranscriptional level via base-pairing with complementary sequences within mRNAs and their interaction plays a key role in the pathogenesis of CRC. There are some differentially expression miRNAs which target genes that exert on various molecular regulation such as SMAD4 targeted by miR-130a/301a/454 cluster [4] and RND3 targeted by miR-17 [5] in proliferation, p70S6K1 targeted by miR-145 [6] in angiogenesis, BCL2 targeted by miR-148a [7] in apoptosis and MMP11 targeted by let-7c [8] in metastasis. They also modulate the balance of resolution of inflammation and prevent tissue damage by regulating the immune response in intestine [9]. A comprehensive meta-analysis of microRNA for predicting colorectal cancer have shown that multiple miRNAs appeared to be more favorable than single miRNA by incorporating 103 studies from 36 articles with a total of 3124 CRC patients and 2579 healthy individuals according to Lin Y et al. [10]. Therefore, it is efficient for choosing the appropriate candidate miRNA and target genes in CRC and discover the novel molecular biomarker combinations validated via public databases and molecular techniques.

**Fig. 1:**
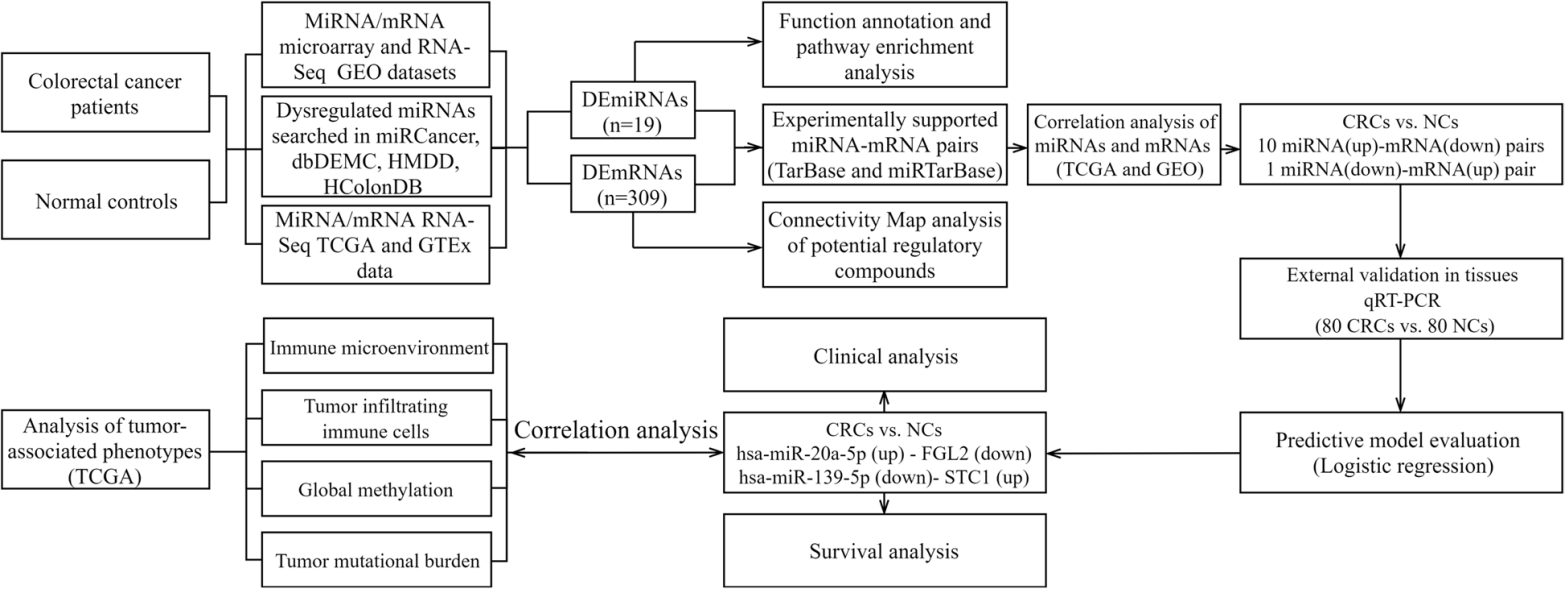
Flow chart for identifying the miRNA-mRNA regulatory pairs and the comprehensive analysis of regulatory pairs role in colorectal cancer (CRC)

By Integrative analysis of paired miRNA-mRNA expression profiles from CRC samples, we identified the miRNA-mRNA regulatory network and their complex roles in CRC pathogenesis especially tumor immunity. An overview of the workflow steps is shown in Fig. 1. In our study, gene and miRNA profiling data were downloaded from The Gene Expression Omnibus (GEO) database, The Cancer Genome Atlas (TCGA) and The Genotype-Tissue Expression (GTEx). To find the pivotal miRNA-mRNA regulatory pairs, we successively conducted differential expression analysis, target gene screening by TarBase and miRTarBase which summarizes experimentally confirmed miRNA-mRNA pairs, function analysis by DAVID-mirPath which is a miRNA pathway analysis web-server and Hiplot tools which is a cloud platform for scientific computation and visualization, and connectivity mapping (cMap) for drug discovery [11]. Then, poly(A) reverse transcriptase quantitative (real-time) polymerase chain reaction (RT-qPCR) assay was performed to detect the expression of miRNAs and target mRNAs in formalin-fixed paraffin-embedded (FFPE) samples, validated using Pearson’s correlation and finally evaluated by logistic regression model. The phenotypic hallmarks provided new insights into miRNA and target-mRNA expression associated with immune microenvironment, tumor infiltrating immune cells, global methylation, tumor mutational burden and RAS gene family mutation status.

Our research performed extensive analysis of miRNA-mRNA regulatory pairs in CRC versus adjacent normal tissue to yield new sights in the underlying mechanism in CRC tumorigenesis. Combination of bioinformatic analysis and qRT-PCR provided with convenience in identifying dysregulated miRNA-mRNA regulatory pairs to improve therapeutic strategies for colorectal cancer patients.

## Results

### Identification of differentially expressed miRNAs (DEMs) and genes (DEGs) in CRC

**Fig. 2:**
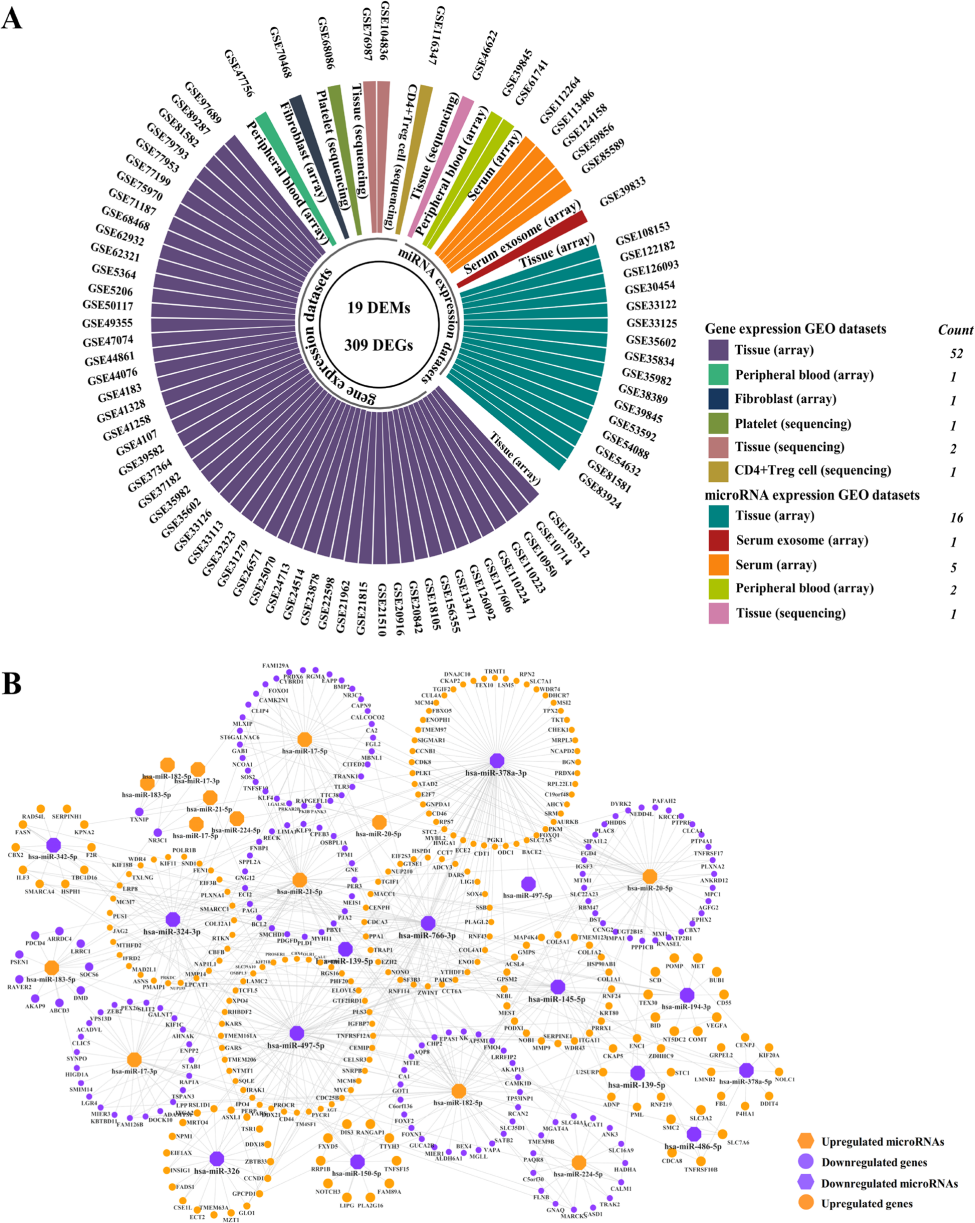
(**A**) The circular-barplot showing the basic information of GEO datasets (GEO accession, source name and experiment type). A total of 83 datasets were included in the study of which 54 were gene expression microarray datasets from tissue, peripheral blood and fibroblast, 4 were gene expression RNA-Seq datasets from tissue, platelet and CD4 + Treg cell, and 25 were miRNA expression datasets including 1 RNA-Seq datasets from tissue and 24 microarray datasets from tissue, peripheral blood, serum and serum exosome. Nineteen DEMs and 309 DEGs were screened in CRCs versus normal controls (NCs). **B** Nineteen microRNAs (miRNAs) to 309 mRNAs network visualized by Cytoscape. There were 250 miRNA (up) – mRNA (down) pairs and 343 miRNA (down) – mRNA (up) pairs screened out by miRtarbase and Tarbase which contain experimentally validated miRNA-mRNA regulatory pairs. Orange dot represents the upregulated miRNAs/mRNAs in CRCs versus NCs, while purple dot represents the downregulated miRNAs/mRNAs in CRCs versus NCs

There were fifty-four gene expression microarray datasets, fifty-two of which from tissue, one from peripheral blood and one from fibroblast. In addition to these, there were also four gene expression RNA-Seq datasets including two datasets from tissue, one from platelet and one from CD4 + Treg cell. A total of twenty-five miRNA expression datasets were filtered out in this study, which consist of one RNA-Seq datasets from tissue and twenty-four microarray datasets from tissue, peripheral blood, serum and serum exosome, respectively. The information of 83 GEO datasets is shown in Table 1. Upregulated and downregulated DEMs/DEGs in CRCs vs. controls were identified using the log2fold change (CRC vs. normal). 19 DEMs and 309 DEGs were the intersection of TCGA, GEO datasets and 3 disease-related miRNA databases (dbDEMC, HMDD and miRcancer) shown in Fig. 2A.

**Table 1:**
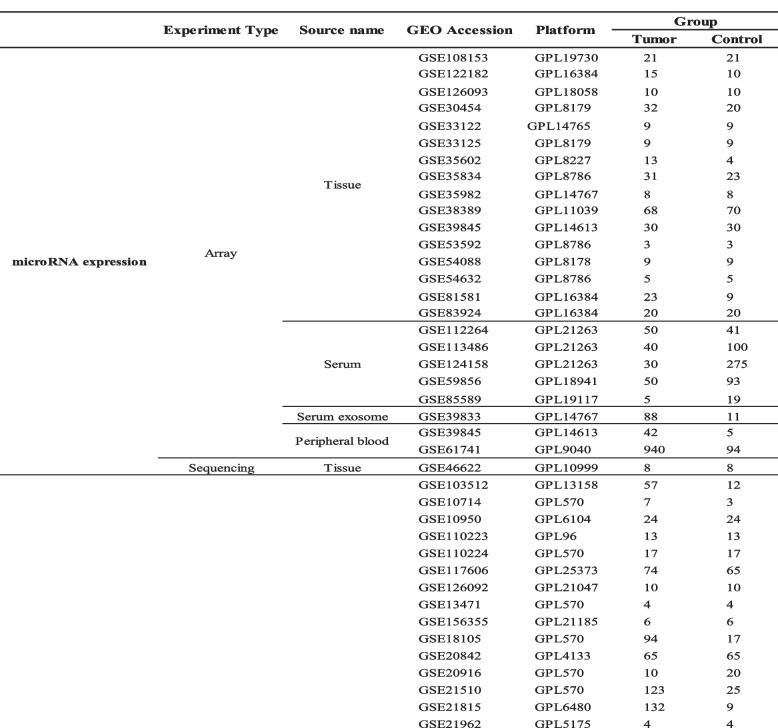

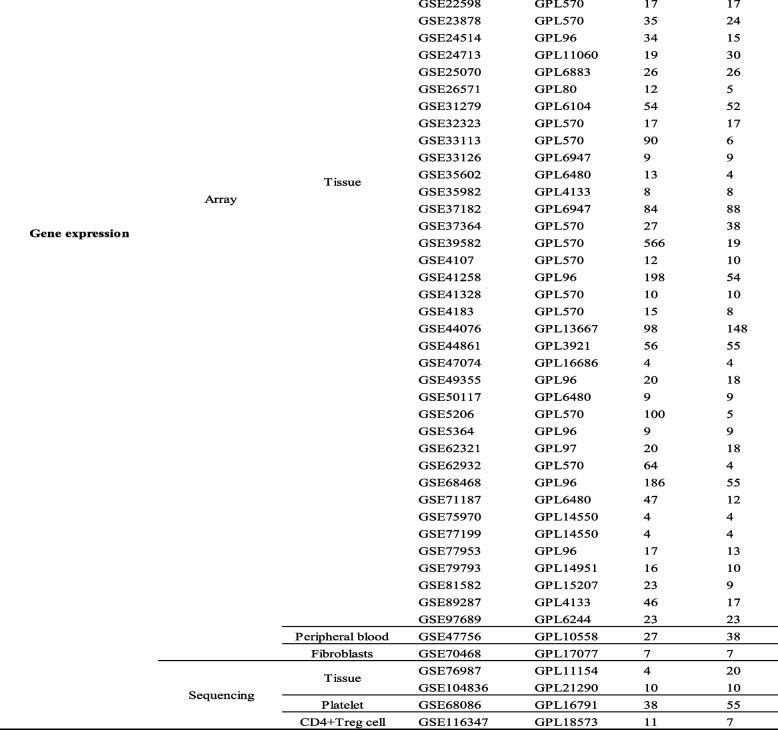
Information pertaining to the selected GEO datasets for colorectal cancer

### Analysis of function enrichment analysis and pathway analysis

**Fig. 3:**
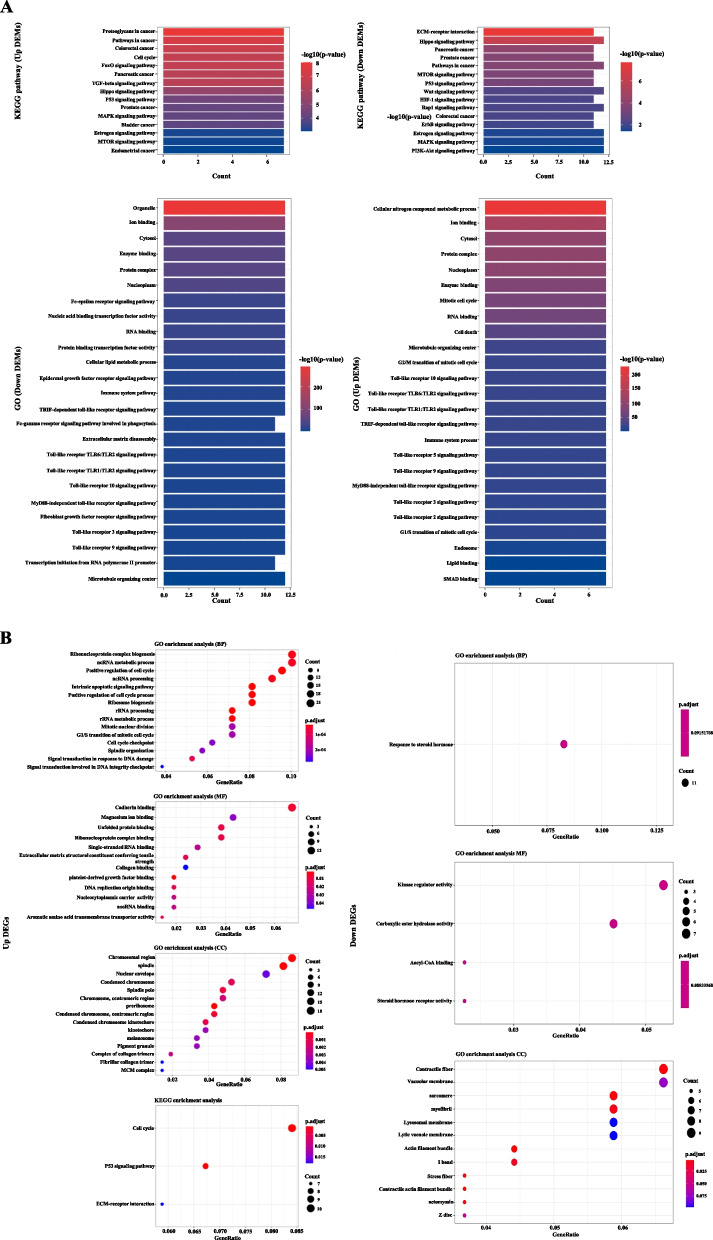
GO and KEGG pathway analysis show the associated function of the target genes of dysregulated miRNAs in CRCs. **A** Left: The top 15 enriched KEGG pathways and the combination of GO terms including the top 15 GO biological processes, 5 cellular components and 5 molecular functions for 7 upregulated miRNAs ordered by -log_10_*P*-value in CRC. Right: The top 15 enriched KEGG pathways and the combination of GO terms including the top 15 GO biological processes, 5 cellular components and 5 molecular functions for 12 downregulated miRNAs ordered by -log_10_*P*-value in CRC. **B** Left: The top 15 enriched GO terms and KEGG pathways for 168 upregulated mRNAs ordered by adjust *p*-value in CRC. Right: The enriched GO terms for 141 downregulated mRNAs ordered by adjust *p*-value in CRC. There is no result for KEGG pathways using “R-clusterProfiler” by Hiplot platform

We performed the enrichment analysis of 7 upregulated miRNAs and 12 down-regulated miRNAs by DAVID-mirPath. Targets of 7 upregulated miRNAs (hsa-miR-17-3p, hsa-miR-17-5p, hsa-miR-182-5p, hsa-miR-183-5p, hsa-miR-20-5p, hsa-miR-21-5p and hsa-miR-224-5p) were enriched in 67 KEGG pathways, 197 Gene Ontology (GO) biological processes, 14 GO celluar components and 21 GO molecular functions listed in Table S2 and the top 15 ordered by -log_10_*P*-value were shown in Fig. 3A. Targets of 12 downregulated miRNAs (hsa-miR-139-5p, hsa-miR-145-5p, hsa-miR-150-5p, hsa-miR-194-3p, hsa-miR-324-3p, hsa-miR-326, hsa-miR-342-5p, hsa-miR-378a-3p, hsa-miR-378a-5p, hsa-miR-486-5p, hsa-miR-497-5p and hsa-miR-766-3p) were enriched in 47 KEGG pathways, 169 Gene Ontology (GO) biological processes, 10 GO cellular components and 12 GO molecular functions listed in Table S3 and the top 15 ordered by -log_10_*P*-value were shown in Fig. 3A. We also analyzed 309 dysregulated genes using clusterProfiler via Hiplot platform separately shown in Fig. 3B.

As a result, the enriched KEGG pathways of dysregulated miRNAs were frequently associated with signal transduction such as Wnt signaling pathway, FoxO signaling pathway, TGF-beta signaling pathway, Hippo signaling pathway, mTOR signaling pathway and MAPK signaling pathway, tumorigenesis such as proteoglycans in cancer, colorectal cancer, pancreatic cancer, prostate cancer and bladder cancer, endocytosis and fatty acid metabolism (full list in Table S3). Lesley M. B et al. have reported the positive association with high plasma levels of fatty acid which may contribute to colorectal carcinogenesis and its increased synthesis capacity on colon cancer risk [12]. Deregulation of these basic biological processes such as catabolic process and cell motility may explain the molecular mechanisms of tumorigenesis in CRC.

### CMap analysis of dysregulated genes in CRC

We employed cMap to find potential compounds that can disturb the dysregulated gene expression pattern. After the query for upregulated tags of 141 genes and down regulated tags of 150 genes which was ordered by adjusted *p*-value in TCGA because of the technical limit of this tool, 67 compounds, of which the significant negative raw connectivity score (nraw_cs) and the significant negative log10Q-value (fdr_q_nlog10) in CRC cell lines (HT1299, HT29 and SW480) were identified as the potential drugs for CRC shown in Fig. S1. According to our analysis, dabrafenib, trametinib and cobimetinib can inhibit the up-regulated genes in CRC. The combination of dabrafenib plus trametinib which is a selective MEK inhibitor has activity in patients of BRAFV600-mutant metastasis CRC [13]. Cobimetinib can inhibit cell proliferation and induce G1 phase arrest and apoptosis in CRC cell lines [14].

CMap mode of action (MoA) for 67 drugs tested in CRC cell lines revealed 38 mechanisms of action shared by the above compounds shown in Fig. S1B. 9 compounds shared the MoA of HDAC inhibitor, 5 compounds shared the MoA of acetylcholine receptor antagonist, 4 compounds shared the MoA as dopamine receptor antagonist, 4 compounds shared the MoA as topoisomerase inhibitor, and 4 compounds shared the MoA as histamine receptor antagonist. PI3K inhibitor, RAF inhibitor, EGFR inhibitor, and MEK inhibitor are shared as the MoA in every 3 compounds.

CMap target analysis revealed 131 drug-target genes shared by 67 compounds shown in Fig. S1B. Nineteen genes are common targets of 16 different compounds-namely, CYP3A4 (3 drugs), KDR (3 drugs), AHR (2 drugs), AKT1 (2 drugs), CDK2 (2 drugs), CHEK1 (2 drugs), CYP2C19 (2 drugs), GSK3B (2 drugs), HRH1 (2 drugs), LCK (2 drugs), MAPK1 (2 drugs), MAPK14 (2 drugs), PDGFRB (2 drugs), PIK3CB (2 drugs), PIK3CG (2 drugs), RAF1 (2 drugs), TOP2A (2 drugs).

We observed similar mechanisms of action among different compounds that can target the dysregulated genes and as the possible therapeutic strategies in CRC.

Screening of negative miRNA/mRNA regulatory pairs associated with CRC .

First, the experimentally validated target mRNAs of 19 differentially expressed miRNAs were selected by miRtarbase and Tarbase. 250 miRNA (up)—mRNA (down) pairs and 343 miRNA (down)—mRNA (up) pairs were screened after intersections of DEGs between 309 DEGs and two databases shown in Fig. 2B. Then, we filtered out 11 miRNA-mRNA pairs with significant negative correlation (adjusted *p*-value < 0.05, adjusted by Benjamini–Hochberg (BH) method) in TCGA listed in Table 2 and full statistical results were listed in Table S4. Then we validated the correlation of 11 miRNA-mRNA pairs including 5 DEMs and 10 DEGs in 6 GEO datasets (GSE35602, GSE41015, GSE126095, GSE33122, GSE81582, GSE128449) listed in Table S5. In addition, we conducted the Kaplan–Meier survival analysis for predicting the prognostic value of these signatures. In the TCGA training set, we built a prognostic classifier using the LASSO Cox regression model, based on the association between the expression of miRNAs and mRNAs and the patients’ overall survival. The partial likelihood deviance (binomial deviance) curve was plotted versus log(λ) through tenfold cross-validation in Fig. S6A and the LASSO coefficient profile of prognostic signatures was plotted in Fig. S6B. Using the LASSO selection model, we built a classifier consists of two miRNA/mRNA negative pairs: hsa-miR-139-5p /STC1 and hsa-miR-20a-5p/FGL2 based on the best lambda (λ) which is 0.0220581.

**Table 2:**
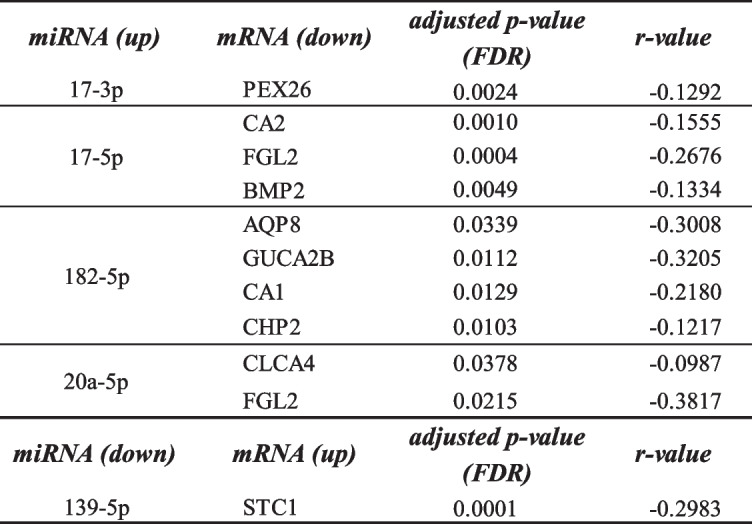
Pearson's correlation analysis of miRNA-mRNA pairs in colorectal cancers in TCGA

### Validation of miRNAs and mRNAs expression in CRC tissues

**Fig. 4:**
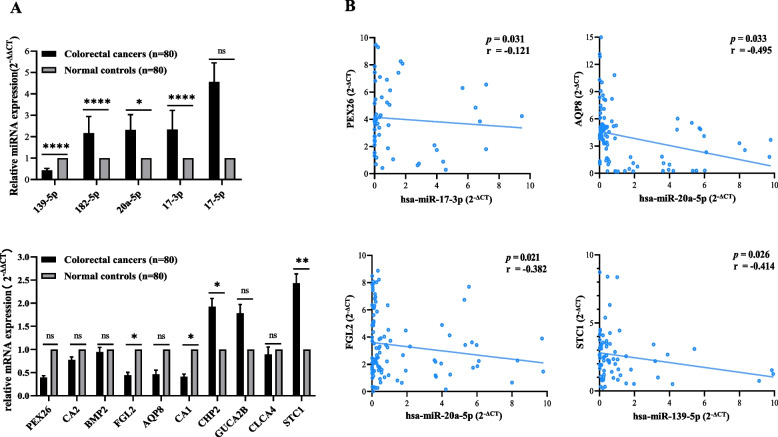
Validating the expression of 5 differentially expressed miRNAs and 11 differentially expressed mRNAs by RT-qPCR. **A** The miRNA expression levels of miR-182-5p, miR-20a-5p, miR-17-3p were upregulated in CRCs, while the miR-139-5p was downregulated in CRCs. The mRNA expression levels of CHP2 and STC1 were upregulated, while FGL2 and CA1 were downregulated in CRCs. **B** Pearson’s correlation analysis of miRNA-mRNA regulatory pairs in 80 paired samples. Four negative correlated miRNA-mRNA regulatory pairs were plotted. Data are presented as mean ± SEM. *p.adj < 0.05, **p.adj < 0.01 and ***p.adj < 0.001 (Student's t-test). *p*-values are listed in Table 3

In order to investigate whether 5 DEMs and 10 DEGs are differentially expressed in CRC versus normal tissues, we analyzed their expression in 80 matched-pairs of tumoral and adjacent normal tissues with ploy(A) RT-PCR. Three significant upregulated miRNAs in CRC were miR-17-3p (FDR < 0.0001, FC = 2.33), miR-182-5p (FDR < 0.0001, FC = 2.16) and miR-20a-5p (FDR = 0.022, FC = 2.31) and only miR-139-5p was downregulated in CRC (FDR < 0.0001, FC = 0.43) shown in Fig. 4A. Two significant downregulated genes in CRC were FGL2 (FDR = 0.017, FC = 0.44) and CA1 (FDR = 0.025, FC = 0.41) and STC1 was significantly overexpressed in CRC (FDR = 0.002, FC = 2.43) also shown in Fig. 4A. We conducted the Pearson’s correlation for interactions of DEMs and DEGs that miR-20a-5p was significant associated with FGL2 (FDR = 0.0215, *r* = -0.3817). We also found the moderately negative correlation between miR-139-5p and STC1 in gene expression level (FDR = 0.0264, *r* = -0.4137) shown in Fig. 4B (see full results in Table 2).

**Table 3:**
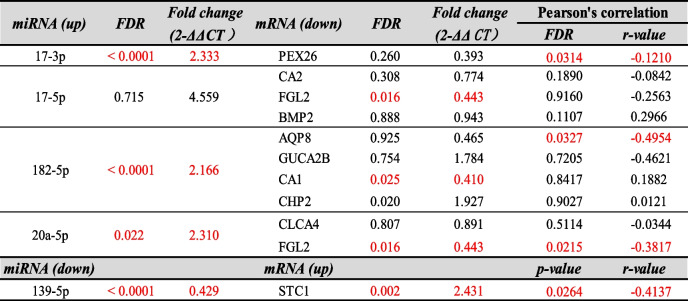
Pearson’s correlation analysis of miRNA-mRNA pairs in FFPE colorectal cancer samples

**Fig. 5:**
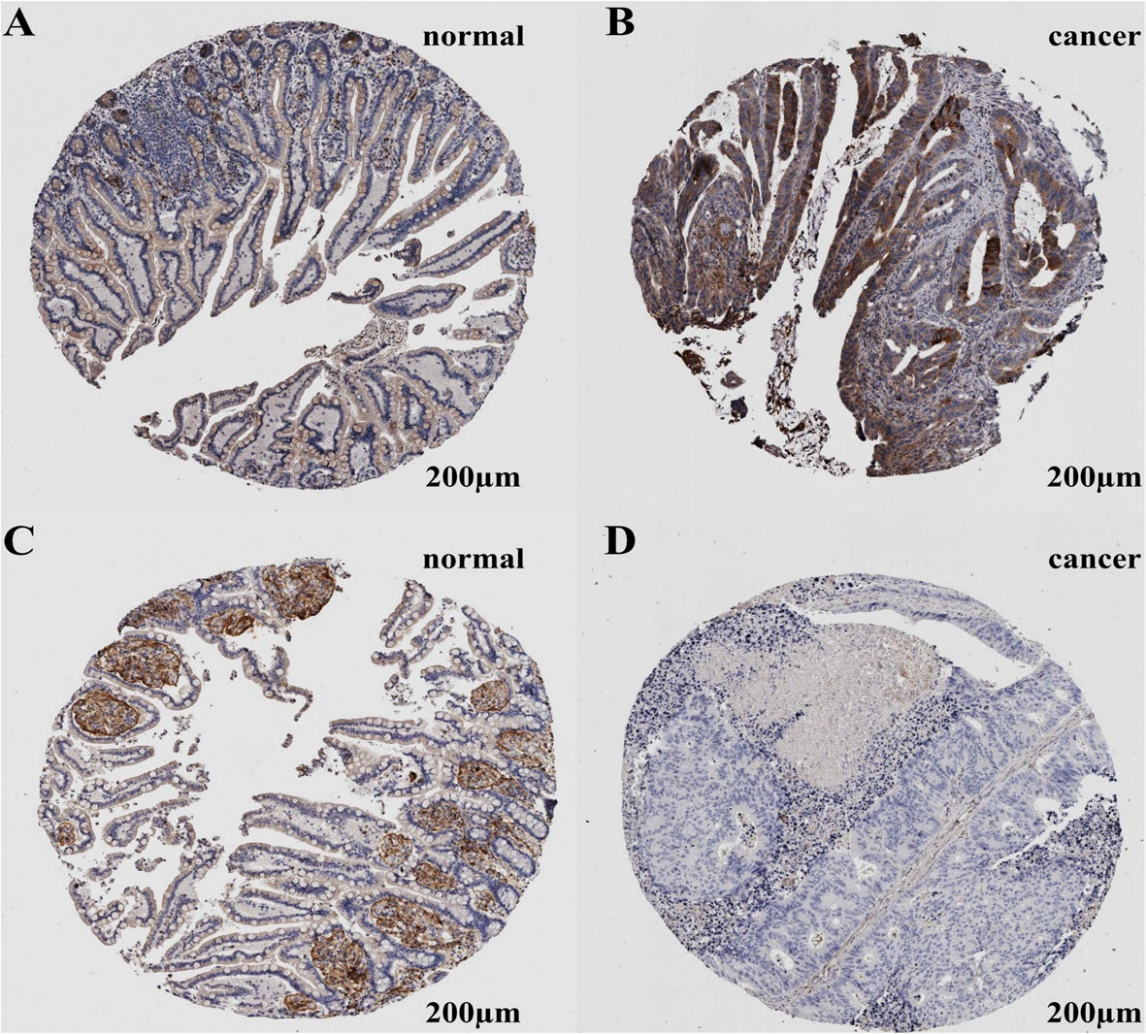
Immunohistochemistry images of STC1 and FGL2 in CRCs and NCs from HPA database. **A** Low immunostaining of STC1 in normal colon endothelial cells (antibody HPA023918); (**B**) Medium immunostaining of STC1 in CRC cells (antibody HPA023918); (**C**) Medium immunostaining of FGL2 in normal colon endothelial cells (antibody HPA026682); (**D**) Immunostaining of FGL2 was not detected in CRC cells (antibody HPA026682)

IHC images in the HPA database evidenced higher expression of STC1 in CRC cells than in normal colonocytes and lower expression of FGL2 in CRC cells than in normal colonocytes shown in Fig. 5.

### Evaluation of predictive value of miRNA-mRNA regulator pairs in CRC

**Fig. 6:**
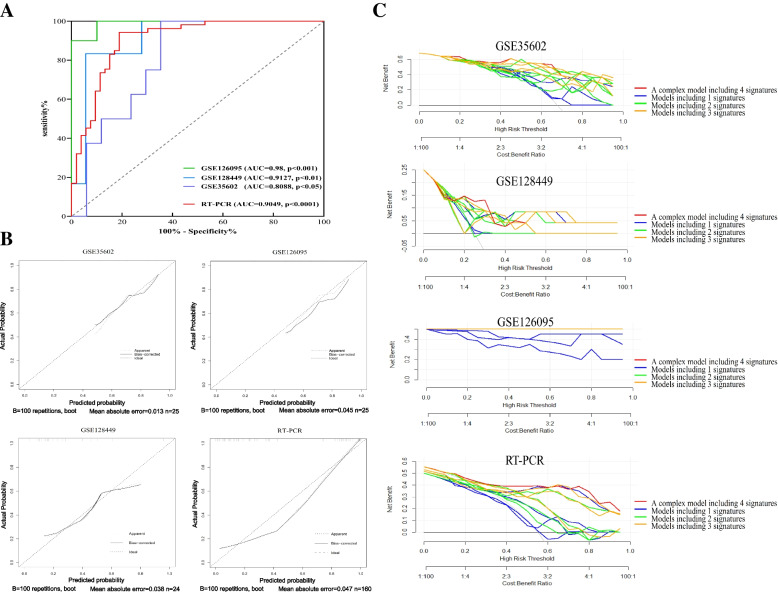
Receiver operating characteristic (ROC) curves, calibration curve and decision curve analysis (DCA) of the complex predictive model including 4 signatures (miR-139-5p, STC1, miR-20a-5p and FGL2) to distinguish CRC samples from normal samples. **A** ROC curves of the complex predictive model in testing datasets GSE126095, GSE128449 and GSE35602, and external validation cohort. **B** Calibration curve of the complex predictive model in testing datasets and validation cohort. **C** The decision curve analysis of 15 models, of which a complex predictive model containing all 4 signatures (miR-139-5p, STC1, miR-20a-5p and FGL2), 4 models containing 3 signatures, 6 models containing 2 signatures and 4 models containing 1 signature

The logistic regression analysis was used to evaluated the predictive value of a panel including 2 miRNA-mRNA regulator pairs: miR-139-5p/ STC1 and miR-20a-5p/ FGL2 in testing cohorts GSE35602, GSE126095 and GSE12844, and validation cohort containing 80 CRC tissues by qRT-PCR. Receiver operating characteristic (ROC) curves, calibration curve and decision curve analysis (DCA) for models were plotted in Fig. 6.

The areas under the curve (AUC) of a complex model (miR-139-5p + STC1 + miR-20a-5p + FGL2) were 0.98 (95% CI: 0.9583 to 1.000, *p* < 0.001) in GSE126095, 0.9127 (95% CI: 0.7972 to 0.9713, *p* = 0.0027) in GSE128449, 0.8088 (95% CI: 0.6402 to 0.9774, *p* = 0.0144) in GSE35602 and 0.9049 (95% CI: 0.8463 to 0.9636, *p* < 0.0001) validated by qRT-PCR shown in Fig. 6A. The calibration curves for the model in 3 testing cohorts and validation cohort were shown in Fig. 6B.

We used DCA to verify the clinical applicability of 15 models, of which 1 model containing all 4 signatures (miR-139-5p, STC1, miR-20a-5p and FGL2), 4 models containing 3 signatures, 6 models containing 2 signatures and 4 models containing 1 signature by quantifying the net benefits at different threshold probabilities. The decision curves in both the external validation cohort and two testing cohorts GSE128449 and GSE35602 showed that the complex model based on 4 signatures (the red line shown in Fig. 6C) could predict the colorectal cancer much better than other 14 models if the threshold probability was between 0–0.60 (in the testing cohort GSE128449, the threshold probability was between 0 and 0.33).

### Association analysis of clinical pathological features and miRNA/mRNA expression level in CRC

Clinical pathological data of CRC patients were summarized in Table 4. The anatomical site of the lesion was in the right colon in the majority of the cases (48, 60%). KRAS mutation was found in almost half of the patients (51.25%) and BRAF mutation was found in 10% of the cases. Because our clinical samples had no enough clinical information, we evaluated miRNAs and mRNAs expression levels in multiple subgroups in TCGA-CRC RNA-Seq data shown in Fig. S3A-F. Receiver operating characteristic (ROC) curve was used to find the best cutoff which was as the basis for grouping of expression levels of miRNAs and mRNAs shown in Fig. S2. MiR-139-5p overexpression was associated with the stage of CRC and age (FDR = 0.0056, FDR = 0.0066, respectively). The expression level of FGL2 was significantly upregulated in microsatellite instable (MSI) CRC versus microsatellite stable (MSS) CRC (FDR = 0.013).

**Table 4:**
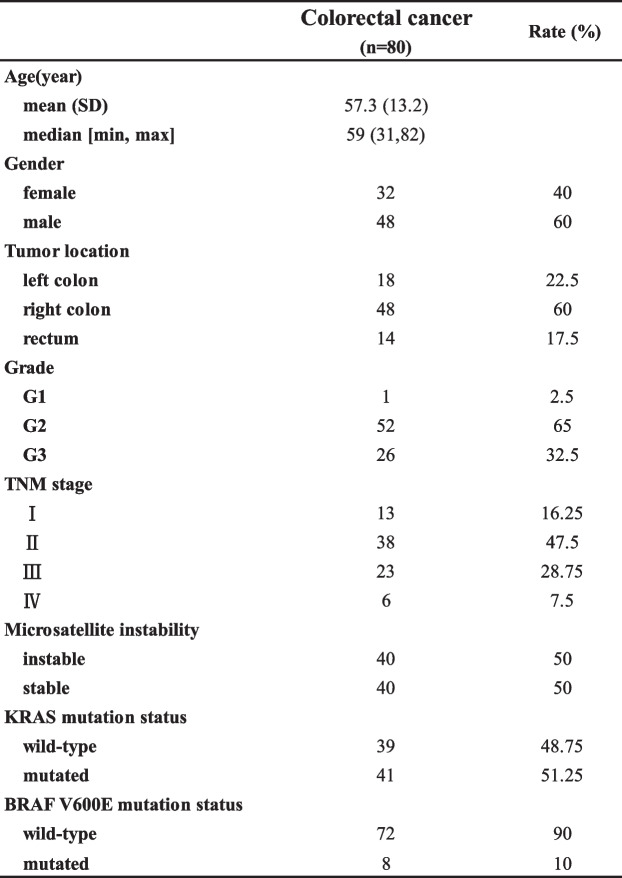
Clinicopathological and molecular features of colorectal cancer patients

We evaluated the association of the expression level of 2 miRNA-mRNA regulatory pairs which were detected to be differentially expressed in CRC versus normal tissue and gene mutations in BRAF gene and 3 Ras family oncogenes HRAS, KRAS and NRAS. The level of miR-139-5p was found to be higher in HRAS wild-type CRC tissues than HRAS-mutated CRC tissues (FDR = 0.030, FC = 3.33). The level of miR-20a-5p was upregulated and FGL2 was downregulated in BRAF wild-type CRC tissues versus BRAF-mutated CRC tissues (FDR = 0.004, FC = 2.05; FDR = 0.026, FC = 0.33, respectively) shown in Fig. S3F (all results listed in Table S6).

As shown in Table S7, human cancer metastasis database (HCMDB) was analyzed that miR-20a-5p by targeting FGL2 and miR-139-5p by targeting STC1 could play a role mainly in liver metastatic CRC.

### Analysis of overall survival

Since our clinical tissue samples and the GEO data had no clinical information, the survival analysis was conducted in TCGA data. The Hazard ratio (HR) of different clinical features in TCGA testing set (*n* = 239) was estimated by univariate and multivariate cox regression analysis. As shown in Table 5 STC1 expression was significantly correlated with the overall survival (OS) with hazard ratio of 1.316 (95% CI: 1.224 to 2.393, *p* = 0.024) in TCGA. The result showed that gender and stage could be as independent risk factors for CRC (HR: 1.649, 95%CI: 1.113 to 3.02, *p* = 0.032; HR: 1.91, 95%CI: 1.422 to 2.813, *p* = 0.011, respectively). We don’t have extra survival data to estimate prognostic model and more research is required about the prognostic value of 2 miRNA-mRNA regulatory pairs in CRC.

**Table 5:**
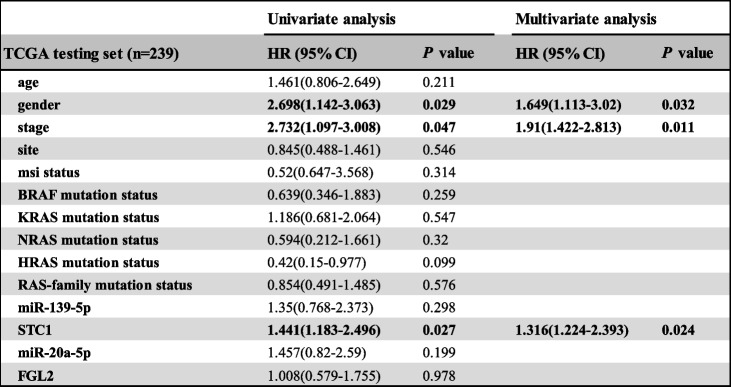
Univariate and multivariate analysis of overall survival in colorectal cancer patients

Kaplan–Meier (K-M) survival for multiple subgroups in TCGA were carried out at least avoiding curves cross. As shown in Fig. S4, the horizontal axis indicated the overall survival time in days, and the vertical axis indicates the survival probability. We found that high expression of STC1 was associated with poor overall survival in patients with colorectal carcinoma.

We also calculated risk scores of each patient in TCGA-COAD based on expression levels and risk coefficients of hsa-miR-139-5p /STC1 and hsa-miR-20a-5p/FGL2 based on the LASSO Cox regression analysis above. The equation Risk score (TCGA testing set) = hsa-miR-139-5p ∗ (-0.10571) + STC1 ∗ (0.01165) + hsa-miR-20a-5p ∗ (0.06809) + FGL2 ∗ (-0.05036) shows the formulae for calculating the risk score for TCGA testing set. The cohort were divided into the high-risk and low-risk group according to the median risk score. We found that colorectal cancer patients in the high-risk group had a shorter overall survival than patients in the low-risk group (log-rank *p* = 0.0007, HR = 2.137, 95%CI: 1.388 to 3.289) shown in Fig. S6C.

### Analysis of tumor-related phenotypes associated with signatures

We applied an established computational method (CIBERSORT) to bulk gene expression profiles of colorectal cancer to infer the proportions of 22 subsets of immune cells. As shown in Fig. S5A, there were 12 types of immune cells differentially expressed in CRC versus control (all results listed in Table S8). We further investigated associations between each cell type and miRNA/ target mRNA expression using Spearman’s correlation. “\” was placed through the cell when padj (BH) value > 0.05 in Fig. S5B-C. The levels of miR-20a-5p and target gene FGL2, and miR-139-5p and target gene STC1 were significantly correlated with M1/M2 macrophages shown in Fig. S5B. MiR-139-5p and target gene STC1 were also associated with activated memory CD4^+^T cells and plasma cells. In our study, 2 miRNA-mRNA regulatory pairs could interact with DNA methylation, tumor immunity and inflammation in the tumor microenvironment shown in Fig. S5C. According to our analysis, the high expression level of FGL2 could lead to the high tumor mutation burden. This result was consistent with the analysis of gene mutation status above that FGL2 was upregulated in MSI CRC and BRAF-mutated CRC shown in Fig. S1.

## Discussion

MicroRNAs which can act as regulators of target genes’ expression and regular biological processes, molecular functions and cellular components had been reported to be critical for the progression of cancers by influencing cell proliferation, cell invasion and tumor metastasis. Combination of miRNAs and mRNAs have a potential clinical value in diagnosis, prognosis and treatment efficacy in colorectal cancer. Identifying the miRNA-mRNA regulatory networks and elucidate their complex roles in immune function, tumorigenesis and molecular mechanisms has a profound meaning.

These regulatory pairs of miRNA-mRNA differed in different cancers presenting the disease-specific expression profiles. Nowadays, various bioinformatics approaches are used as screening tools to identify miRNA-mRNA regulatory pairs such as computational target prediction. Accurately predicting miRNA targets remains challenging due to factors such as imperfect sequence specificity, target site availability and the thermodynamic stability of the structure of mRNA itself [15]. In our research, we conducted the Pearson’s correlation analysis of the miRNAs and target mRNAs in six GEO datasets which examined the expression values of miRNAs and mRNAs from the same patient shown in the Table S5. We found the correlation coefficients of two negative miRNA-mRNA regulatory pairs were low in FFPE tissue samples by RT-qPCR. One reason is perhaps that repeated washing, centrifugation, purification and other steps can cause a considerable amount of nucleic acid loss and increase the possibility of nucleic acid fragmentation and hydrolysis. The half-live of mRNAs was 16.4 h which were shorter than circRNAs (24.56 ± 5.2 h), lncRNAs (17.46 ± 3.0 h) and miRNAs (16.42 ± 4.2 h) in blood samples by Chong.W et al. [16]. The coding DNA sequence length, %GC content, and 3’UTR length were found that might be associated with the transcript degradation rates by Romero et al. [17]. The findings of the Li et al.were that cytidine-containing poly(A) tails can substantially enhance the protein production rate and duration of synthetic mRNAs [18]. Another reason may be that the Pearson correlation captures only linear dependency between expression of mRNA and miRNA. The correlation network between miRNA and mRNA can be more complex. We screened the target genes of potential DE-miRNAs using TarBase and miRTarBase, which contain experimentally verified miRNA/ target-gene pairs. The direct interaction between hsa-miR-139-5p and STC1 is validated using crosslinking immunoprecipitation (CLIP), coupled with high throughput sequencing (HITS-CLIP) by Karginov FV et al. [19]. The direct interaction between hsa-miR-20a-5p and FGL2 is validated using photoactivatable ribonucleoside enhanced CLIP (PAR-CLIP) by Gottwein, Eva et al. [20]. There are other experimental procedures using high-throughput sequencing for verifying the authenticity of the identified miRNA-mRNA regulatory pairs such as crosslinking, ligation, and sequencing of hybrids (CLASH), biotin-Microarrays and western blot. Alternatively, there are additional methodologies that DIANA-microT-CDs [21] is based on PAR-CLIP data and DeepMirTar tool [22] is based on stacked de-noising auto-encoders at the site level, LeMoNe [23] which are similarity-based methods contains the high intrinsic correlation between miRNA and mRNA, and DIABLO [24] is built on canonical correlation analysis (CCA) [25] which describes the strength of the linear dependence in terms of the best low-dimensional linear projections of two variables, matrix factorization such as the Joint and Individual Variation Explained (JIVE) [26], Multi-Omics Factor Analysis (MOFA) [27], and the Independent component analysis (ICA) [28]. Therefore, it is essential that the comprehensively and precisely dissection of miRNA-mRNA associations need to combine results obtained by different methodologies such as miRNA targets identification, experimentally validated miRNA targets databases, miRNA targets prediction based on binding sites and deep learning algorithms developing for integration of miRNA and mRNA expression data. Despite strengths and weaknesses characterizing each strategy, the accuracy of these prediction tools can be improved only by obtaining more experimentally validated expression profile data. Identifying the miRNA-mRNA regulatory pairs which have good accuracy will aid to deepen the understanding of miRNA functions in tumor development and tumorigenesis.

## Conclusions

We performed extensive analysis of miRNA-mRNA regulatory pairs in CRC versus adjacent normal tissue. In 83 GEO datasets, the expression profiles of miRNAs and mRNAs were screened using GEO2R, “R-limma” and “R-edgeR”. Then, combination of TCGA data and GTEx data from normal tissues was used to identify the candidate DEMs and DEGs which were compared with results of 4 cancer-related databases (miRCancer, dbDEMC, HMDD and HColonDB) simultaneously. Through the muti-step method, 19 differentially expressed miRNAs and 309 differentially expressed mRNAs were identified.

Function analysis and cMap analysis were conducted that candidate DEMs and DEGs which were screened from TCGA, GEO, GTEx and 4 databases were associated with classic cancer-related signaling pathways such as Wnt signaling pathway, TGF-beta signaling pathway and mTOR signaling pathway. Notably, fatty acid metabolism which is the enriched KEGG pathways has been paid enough attention to so far. High-fat diet (HFD) which is risk factor for cancers promotes regeneration capacity and tumorigenesis by enhancing intestinal stem cell (ISC) located at the base of intestinal crypts and cell proliferation [29]. The findings of MEK inhibitors trametinib and cobimetinib, and BRAFV600-mutatant-related dabrafenib considered as therapeutic strategies of cancers demonstrated the importance of 309 dysregulated genes in colorectal cancer.

In this study, we adopted the strict criterion to identify vital miRNA-mRNA regulatory pairs. Briefly, the target mRNA of DEMs should be differentially expressed, significant negative correlated with DEM regulator by Pearson’s correlation, and further validated by two databases containing experimentally validated miRNA-target interactions, miRTarbase and Tarbase. At first, 250 miRNA (up)—mRNA (down) pairs and 343 miRNA (down)—mRNA (up) pairs were screened after intersections of DEGs between 309 DEGs and two databases shown in Fig. 2B. Then, we filter out 11 significantly negative correlated miRNA-mRNA pairs (adjusted *p*-value < 0.05) which were also estimated in 6 GEO datasets listed in Table 2 and Table S5. We further detected the expression level of 11 miRNA-mRNA regulatory pairs in 80 pairs FFPE colon tissues by poly(A) qRT-PCR.

Ultimately, two pivotal negative correlated miRNA-mRNA regulatory pairs (miR-20a-5p/ FGL2 and miR-139-5p/ STC1) were considered for inclusion in the logistic regression model. The following analysis will support the predictive value of miRNA-mRNA pairs. On one hand, receiver operating characteristic (ROC) curve was used to evaluate a total of 15 randomly combinations of 4 signatures and calibration curve was used to estimate the calibration performance of the complex model including 4 signatures. On other hand, we used decision curve analysis (DCA) to evaluate clinical utility of 15 models. The complex model (miR-139-5p + STC1 + miR-20a-5p + FGL2) was the best predictive model when compared with other 14 combinations in 2 testing cohorts GSE35602 and GSE12844, and the validation cohort containing 80 CRC tissues. Some researchers have reported the functional role for miR-139-5p in breast cancer cell motility and invasion [30]**,** in colorectal cancer epithelial-mesenchymal transition [31] and cell proliferation [32], and in cervical cancer cell proliferation and migration [33]. Compared with previous studies on the miRNA expression profile of CRC, it has the possibility to serve as a molecular therapeutic target and prognostic marker in colorectal cancer (CRC) [34], tongue squamous cell carcinoma (TSCC) [35], breast cancer (BC) [36], glioblastoma multiforme (GBM) [37] and non-smalll cell lung cancer (NSCLC) [38]. MiR-20a-5p belongs to the miR-17–92 cluster which is reported to be overexpressed in hepatocellular carcinoma (HCC) [39], triple-negative BC [40], renal cell carcinoma (RCC) [41] and CRC in many studies including our research. According to recent studies, it is also linked to cell proliferation, activation of monocyte/macrophage lineage, B cells, Th1, Th2, Th17 and TFH cells in innate and adaptive immunity [42]. FGL2, MAPK-mediated upregulation of fibrinogen-like protein 2, was downregulated in CRC tissues. The knockdown of FGL2 can reduce the proliferation, migration and invasion in CRC cell lines [43]. STC1, secreted glycoprotein stanniocalcin-1, is the mediator of metastasis by platelet-derived growth factor (PDGF) related to cancer-associated fibroblasts (CAF) in CRC [44].

We summarized the clinical pathological features in TCGA and miR-139-5p was found to be differentially expressed in stage I-II versus stage III-IV, while the high expression of FGL2 was associated with microsatellite instability in CRC. HRAS mutation status and BRAF mutation status were confirmed to interact with the expression level of miR-139-5p and FGL2, respectively. In metastasis CRC versus controls, two pivotal negative correlated miRNA-mRNA regulatory pairs (miR-20a-5p/ FGL2 and miR-139-5p/ STC1) could be considered to associate with tumor metastasis in CRC. High STC1 expression is a significant independent predictor of poor survival in colorectal cancer by SHUZO T et al. [45]. Although STC1 wasn’t correlated with the OS of CRC in validation set (*n* = 71) (HR: 1.025, 95%CI: 0.468 to 2.244, *p* = 0.952) and Kaplan–Meier (K-M) survival curves didn’t give any indication about the influence of STC1 expression in overall survival, we couldn’t rule out the prognostic value of STC1. However, a detailed analysis could not be performed due to insufficient information and there is ambiguity in the prognostic value of two miRNA-mRNA regulatory pairs in CRC. MiR-20a-5p by targeting FGL2 and miR-139-5p by targeting STC1 could have an impact on tumor microenvironment by interacting with tumor-related inflammation and infiltration of macrophages and CD4^+^T cells. Especially, FGL2 which was upregulated in MSI CRC and BRAF-mutated CRC could lead to high tumor mutation burden.

## Methods

### Data acquisition and processing of miRNA and gene expression profiles

The Cancer Genome Atlas (TCGA) colon adenocarcinoma (COAD) and rectal adenocarcinoma (READ) miRNA and mRNA sequencing expression profiles and associated clinicopathological data were downloaded from the GDC data portal at the National Cancer Institute (https://portal.gdc.cancer.gov/). There is no apparent difference between colon and rectal samples validated by Principal component analysis (PCA) and merging samples is no need to adjust [46]. So, we combined TCGA-COAD and TCGA-READ samples into a single colorectal adenocarcinoma (COADREAD or CRC) cohort. GTEx data were obtained from UCSC Xena browser which is a combined cohort of TCGA, TARGET and GTEx samples (https://xenabrowser.net/datapages/). We processed the data from GTEx and TCGA using perl. A total of 453 tumor tissue samples, 41 normal samples from TCGA and 308 normal samples from GTEx were included in this article. Then we searched colorectal cancer relevant gene microarray expression datasets and high-throughput sequencing expression datasets from the Gene Expression Omnibus (GEO) database (http://www.ncbi.nlm.nih.gov/geo/) with the following keywords: “colorectal cancer”. Filters were set to “series” and “Expression profiling by array”, “Expression profiling by high-throughput sequencing”, “Non-coding RNA profiling by array”, “Non-coding RNA profiling by high-throughput sequencing” and “Homo sapiens”. We also collected differentially expressed miRNAs in 3 databases: miRCancer [47], Database of Differentially Expressed MiRNAs in human Cancers (dbDEMC) [48], Human MicroRNA Disease Database (HMDD) [49] and genes in Human Colon cancer Database (HColonDB) [50]. RNA-Seq data were analyzed by “edgeR” R package. The differentially expressed genes (DEGs) and differentially expressed miRNAs (DEMs) were obtained from microarray expression profiles using the web analysis tool GEO2R, which is used to compare groups of samples by the GEOquery and limma R packages from the Bioconductor project in the GEO database (http://www.ncbi.nlm.nih.gov/geo/geo2r/). The cut-off criteria were adjusted *p*-value (FDR) < 0.05 and |log2 (fold change) |≥ 1.

Identification and function analysis of miRNA/target-gene pairs .

Firstly, we screened the target genes of potential DE-miRNAs using TarBase and miRTarBase, which contain experimentally verified miRNA/ target-gene pairs. Then the expression correlation between miRNA-mRNA with negative correlations identified from the above databases was evaluated using Pearson’s correlation. Visualization of the miRNA-mRNA negative regulatory network was conducted using Cytoscape software (v3.8.0) [51]. Gene ontology (GO) functional analysis and a Kyoto Encyclopedia of Genes Genomes (KEGG) pathways analysis [52] against the DEGs and DEMs in the network were performed by using DAVID-mirPath which is a miRNA pathway analysis web-server [53] and the clusterProfiler tool in Hiplot (https://hiplot.com.cn), a comprehensive web platform for scientific data visualization [54]. Adjusted *p*-value (FDR) < 0.05 was considered to indicate a statistically significant difference of enriched GO/KEGG terms.

### Connectivity map analysis of potential compounds capable of targeting the differentially expressed genes

We employed the Connectivity Map (cMap) analysis by querying dysregulated genes (at least 10 genes) in colorectal cancers versus normal controls for discovering candidate compounds that might target pathways related to CRC via clue.io software platform (https://clue.io/query). The normalized connectivity score (norm_cs) ranging from -3 to 3 was used to estimate the closeness between up-regulated genes and compounds. The positive score ranging from 0 to 3 indicated a promotive effect of compound on the up-regulated genes. Negative log10 transformed FDR q-values (fdr_q_nlog10) > 2 was set as the filter condition.

### Survival analysis

Univariate and multivariate Cox regression analyzed by “survival” package (http://cran.r-project.org/web/packages/survival/index.html) [55]. The hazard ratio (HR) and 95% confidence interval (CI) were estimated. Kaplan–Meier method was also used to calculate overall survivals, and the log-rank test analyzed the differences. *P* value < 0.05 was the significant cutoff.

### Evaluation of interactions of miRNA-mRNA pairs and tumor-relative phenotypes and gene mutation status

The fraction of 22 infiltrating immune cell types was calculated using CIBERSORT, a gene-based deconvolution algorithm (https://cibersort.stanford.edu/index.php/) [56]. The differences of these immune cells between TCGA-CRCs and normal controls were compared via the Wilcoxon rank-sum test. ESTIMATE software based on the mRNA-seq data was used to estimate the stromal and immune levels for TCGA-CRC samples [46]. The methylation levels of the CpG sites in TCGA-COAD and TCGA-READ samples were obtained using UCSC Xena platform (https://xena.ucsc.edu/) detected by the Illumina Infinium HumanMethylation450 BeadChips platform, which covered 485,577 CpG loci. The sum of the methylation levels of all 485,577 CpG sites in each sample was calculated as overall DNA methylation level. Tumor mutational burden (TMB) was used to measure the total number of somatic variants per megabase (MB) of the genome. Masked Somatic Mutation data (varscan. Somatic. Maf) were obtained using the “maftools”in R package [57]. We used 38 Mb as the estimate of the exome size. TMB estimate for each sample is equal to the total mutation frequency/38. TCGA-CRC samples were grouped into wild-type and mutated in RAS genes family KRAS, HRAS, NRAS and BRAF based on TCGA-COAD and TCGA-READ somatic mutation datasets obtained from UCSC Xena.

### Sample collection and RNA isolation

80 paired formalin-fixed paraffin-embedded (FFPE) CRC tissues and corresponding adjacent normal tissues were obtained from patients who underwent surgery at the First Affiliated Hospital of Nanjing Medical University. All samples used in this study were collected with patients’ consent. The present study has been approved by the institutional ethics committee and the patients written informed consent has been obtained (ID: 2016-SRFA-148). The clinical characteristics of the 80 colorectal cancer patients are showed in Table 4. Total RNA was extracted from FFPE tissues using RNAprep Pure FFPE Kit (TIANGEN) according to the manufacturer’s instructions. RNA concentrations were measured with NanoDrop ND-1000 spectrophotometer (Thermo Fisher Scientific).

### Quantitative reverse transcription PCR (qRT-PCR) assay

Selected DEGs and DEMs were verified by qRT-PCR using PrimeScript RT reagent Kit (Takara) and SYBR Premix Ex Taq II (Takara) after adding a poly(A) tail to RNA by Poly(A) Polymerase Kit (Takara). All kits were used according to the manufacturer's protocol. The PrimeScript RT reagent Kit and SYBR Premix Ex Taq II kit contain the commercial Uni-RT primer and Uni-Reverse primer. The PCR reactions were carried out in final volumes of 10 μL on the qTOWER^3^ 84 (Analytik Jena) at 95 °C for 20 s, followed by 40 cycles of 10 s at 95 °C, 20 s at 60 °C. The sequences of PCR primers are listed in the Table S1. RUN6B (U6), GAPDH and 18S rRNA were considered as reference genes for normalization, and the comparative cycle threshold (2^−△△Ct^) method was used to analyze the relative expression of miRNAs and genes by Livak KJ et al. [58].

### Statistical analysis

IBM SPSS Statistics v.26 software (IBM Corporation, Armonk, NY, USA), R language v3.6.3 (https://cran.r-project.org/) or GraphPad Prism software were used to analyze the data. Continuous variables were reported using the mean and standard deviation (SD). Student’s t-tests were performed, and *p*-values and adjusted *p*-values were calculated. MiRNA and gene with a |log2FC|> 0.58, *P* < 0.05 and FDR (False Discovery Rate) < 0.05were considered to be statistically significant. The association between the expression of miRNAs and genes was analyzed by Pearson’s correlation in MSI and MSS CRC tissues. The predictive value of miRNA-mRNA pairs was assessed by the area under the ROC curves (AUC) which is used to evaluate the discrimination of the model, and calibration curve which is used to evaluate the accuracy of the model. *P* < 0.05 was considered statistically significant. Decision curve analysis (DCA) based on Logistic regression is used to verify the clinical applicability of the model. Pearson’s correlation method was used to calculate correlation between DEGs or DEMs and all tumor-related phenotypes. OS was defined as the interval from surgery to the date of death. Survival curves plotted by the Kaplan–Meier method were analyzed by the log-rank test and *p* < 0.05 was regarded as statistically significant. Cox regression analyses were performed and the hazard ratio (HR) and 95% confidence interval (CI) were calculated to identify statistically significant DEGs or DEMs (*p*-value < 0.05) associated with survival. We draw plots using R v3.6.3, GraphPad Prism and Hiplot software.

### Supplementary Information

**Additional file 1: Fig. S1.** Connectivity map potential compounds and mechanisms of action analysis of differentially expressed genes based on clue.io software platform.** Fig. S2.** ROC curves for miRNAs and target mRNAs to calculate the best cutoff value for the clinical pathological features analysis and survival analysis of colorectal cancers.** Fig. S3.** MiRNAs and target mRNAs expression level analysis in subgroups based on clinical pathological features of colorectal cancer patients in TCGA.** Fig. S4.** Kaplan-Meier survival analysis for differentially expressed miRNAs and target mRNAs in colorectal cancer.** Fig. S5.** The association between immune-related phenotypes and miRNAs/ target mRNAs expression levels in colorectal cancer.** Fig. S6.** Potential prognostic predictors selection using LASSO Cox regression model and Kaplan-Meier survival curves for CRC patients with high-risk group and low-risk group which show statistically significant difference.

**Additional file 2: Table S1.** The sequences of primers for candidate miRNAs and targeted mRNAs.** Table S2.** Function annotation and pathway enrichment analysis of 7 upregulated microRNAs.** Table S3.** Function annotation and pathway enrichment analysis of 12 downregulated microRNAs.** Table S4.** Pearson’s correlation of miRNAs and mRNAs which were screened from 2 databases (miRTarBase and TarBase) containing experimentally validated miRNA-mRNA regulatory pairs.** Table S5.** Pearson’s correlation analysis of the screened miRNA-mRNA pairs validated in 6 GEO datasets.** Table S6.** Analysis of microRNAs and mRNAs expression level in 5 subgroups based on 4 genes mutation status.** Table S7.** Expression analysis for DEMs and DEMGs in metastatic colorectal cancer by HCMDB.** Table S8.** Analysis of CIBERSORT scores of 22 types of immune cells in colorectal cancers versus controls.

## Data Availability

The datasets generated during and/or analysed during the current study are available from the corresponding author on reasonable request.
