# Supplementary material for: Comprehensive analysis of miRNA-mRNA regulatory pairs associated with colorectal cancer and the role in tumor immunity
Source: BMC Genomics. 2023 Nov 30;24:724. doi: 10.1186/s12864-023-09635-4 (PMC10688136; doi:10.1186/s12864-023-09635-4)
Supplement: Supplementary file 1 — Additional file 1: Fig. S1. Connectivity map potential compounds and mechanisms of action analysis of differentially expressed genes based on clue.io software platform. Fig. S2. ROC curves for miRNAs and target mRNAs to calculate the best cutoff value for the clinical pathological features analysis and survival analysis of colorectal cancers. Fig. S3. MiRNAs and target mRNAs expression level analysis in subgroups based on clinical pathological features of colorectal cancer patients in TCGA. Fig. S4. Kaplan-Meier survival analysis for differentially expressed miRNAs and target mRNAs in colorectal cancer. Fig. S5. The association between immune-related phenotypes and miRNAs/ target mRNAs expression levels in colorectal cancer. Fig. S6. Potential prognostic predictors selection using LASSO Cox regression model and Kaplan-Meier survival curves for CRC patients with high-risk group and low-risk group which show statistically significant difference. [file 12864_2023_9635_MOESM1_ESM.docx]

**Fig. S1**

**
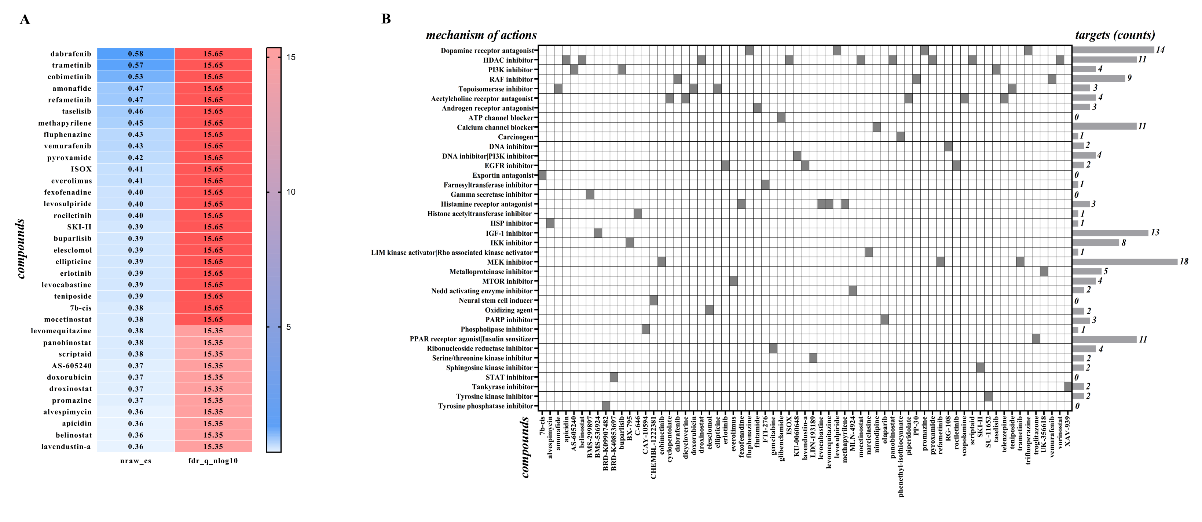
**

Connectivity map potential compounds and mechanisms of action analysis of differentially expressed genes based on clue.io software platform.

**Fig. S2**

**
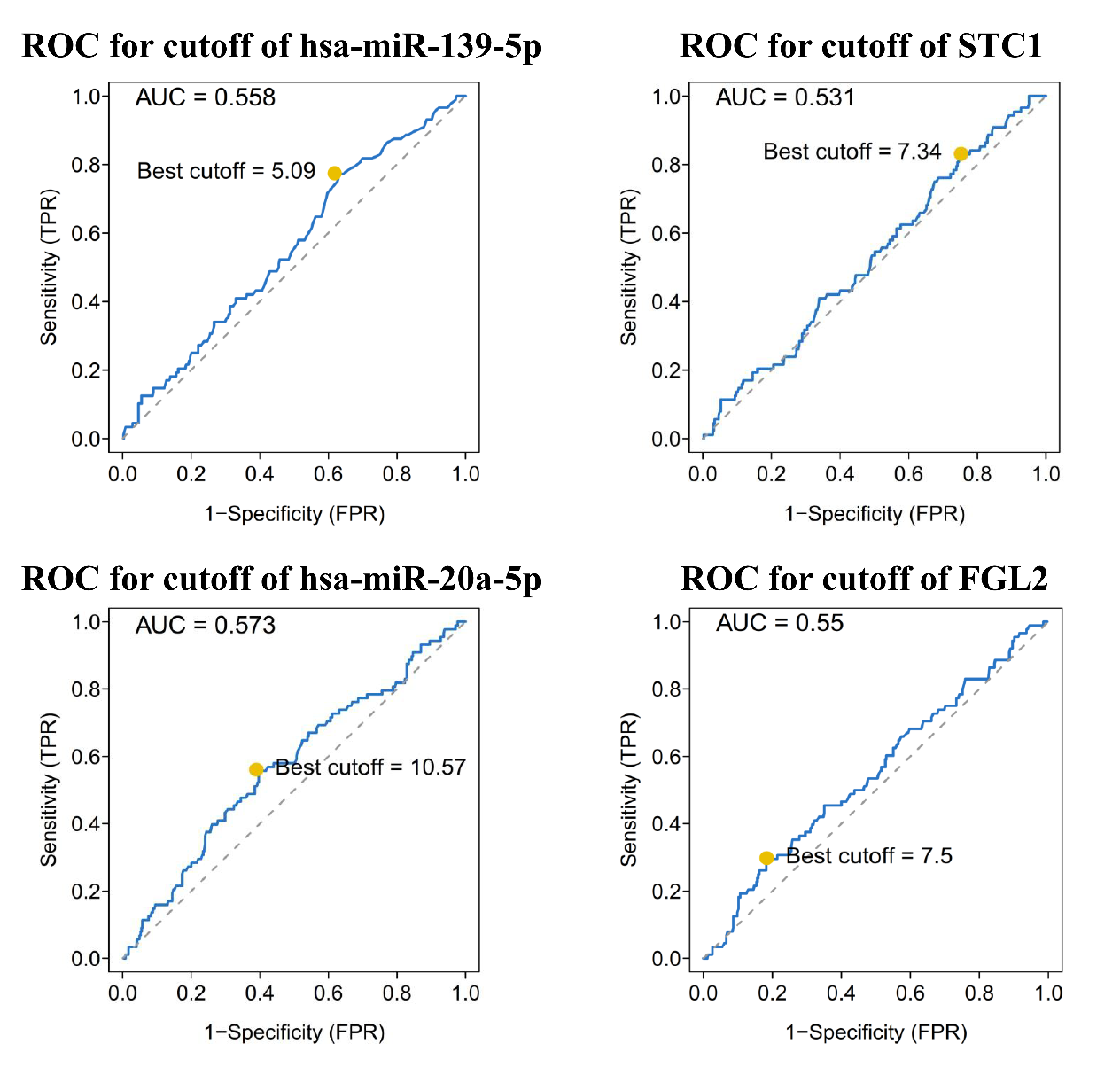
**

ROC curves for miRNAs and target mRNAs to calculate the best cutoff value for the clinical pathological features analysis and survival analysis of colorectal cancers.

**Fig. S3**

**
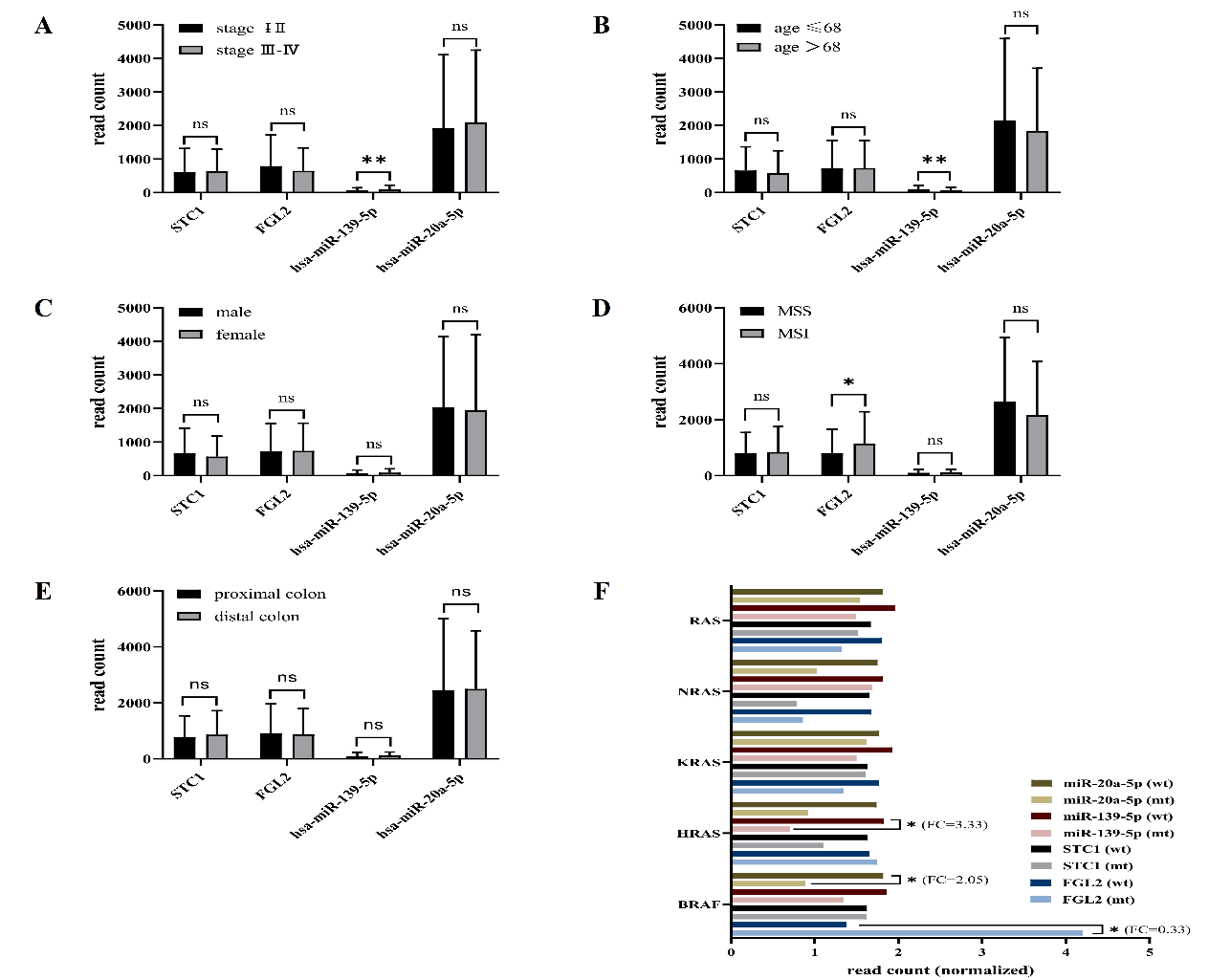
**

MiRNAs and target mRNAs expression level analysis in subgroups based on clinical pathological features of colorectal cancer patients in TCGA.

**Fig. S4**

**
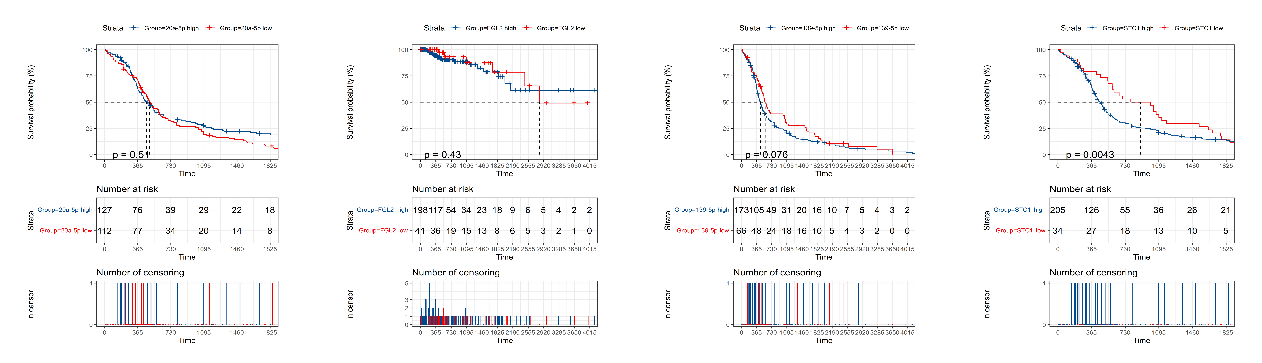
**

Kaplan-Meier survival analysis for differentially expressed miRNAs and target mRNAs in colorectal cancer.

**Fig. S5**

**
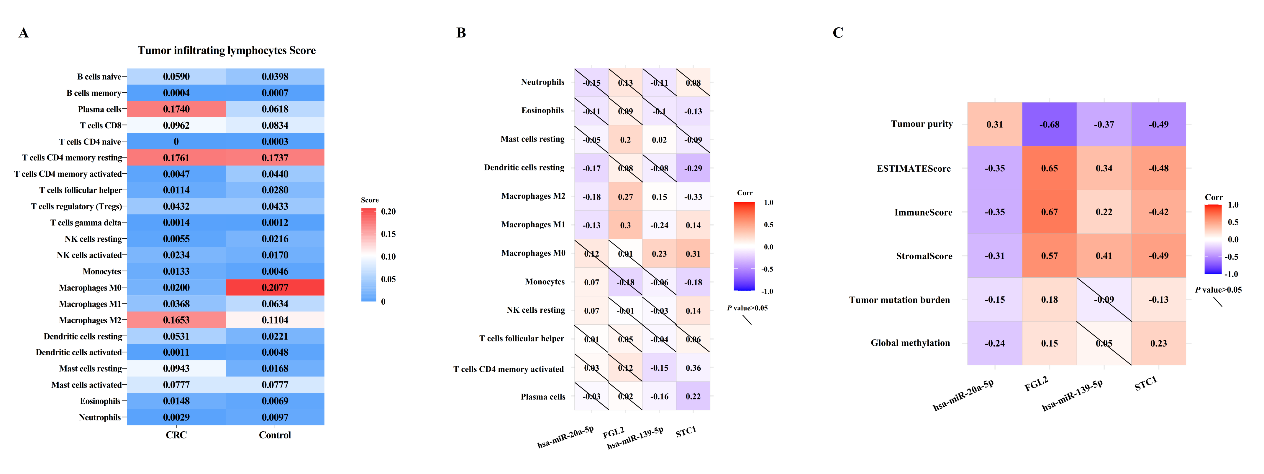
**

The association between immune-related phenotypes and miRNAs/ target mRNAs expression levels in colorectal cancer.

**Fig. S6**


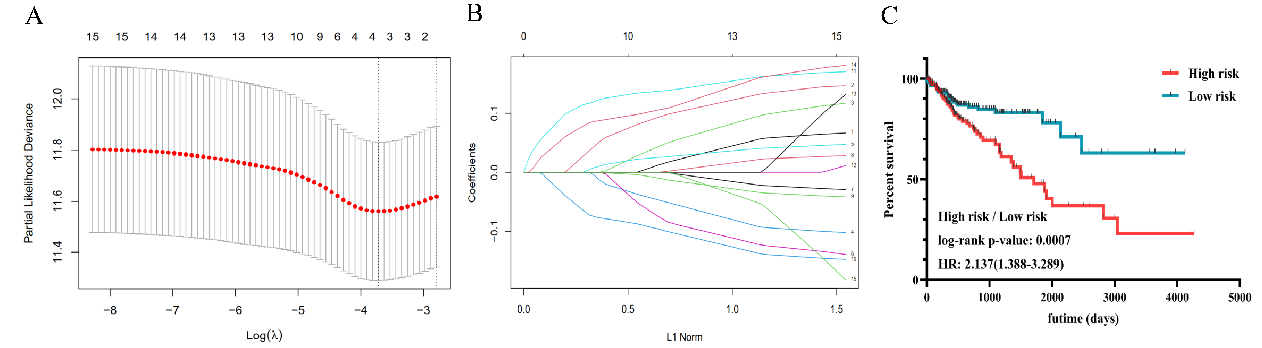


Potential prognostic predictors selection using LASSO Cox regression model and Kaplan-Meier survival curves for CRC patients with high-risk group and low-risk group which show statistically significant difference.
