# Supplementary material for: Comprehensive analysis of miRNA-mRNA regulatory pairs associated with colorectal cancer and the role in tumor immunity
Source: BMC Genomics. 2023 Nov 30;24:724. doi: 10.1186/s12864-023-09635-4 (PMC10688136; doi:10.1186/s12864-023-09635-4)
Supplement: Supplementary file 2 — Additional file 2: Table S1. The sequences of primers for candidate miRNAs and targeted mRNAs. Table S2. Function annotation and pathway enrichment analysis of 7 upregulated microRNAs. Table S3. Function annotation and pathway enrichment analysis of 12 downregulated microRNAs. Table S4. Pearson’s correlation of miRNAs and mRNAs which were screened from 2 databases (miRTarBase and TarBase) containing experimentally validated miRNA-mRNA regulatory pairs. Table S5. Pearson’s correlation analysis of the screened miRNA-mRNA pairs validated in 6 GEO datasets. Table S6. Analysis of microRNAs and mRNAs expression level in 5 subgroups based on 4 genes mutation status. Table S7. Expression analysis for DEMs and DEMGs in metastatic colorectal cancer by HCMDB. Table S8. Analysis of CIBERSORT scores of 22 types of immune cells in colorectal cancers versus controls. [file 12864_2023_9635_MOESM2_ESM.docx]

**Supplementary tables**

**Table S1: The sequences of primers for candidate miRNAs and targeted mRNAs**

**Table S2: Function annotation and pathway enrichment analysis of 7 upregulated microRNAs**

**Table S3: Function annotation and pathway enrichment analysis of 12 downregulated microRNAs**

**Table S4: Pearson’s correlation of miRNAs and mRNAs which were screened from 2 databases (miRTarBase and TarBase) containing experimentally validated miRNA-mRNA regulatory pairs**

**Table S5: Pearson’s correlation analysis of the screened miRNA-mRNA pairs validated in 6 GEO datasets**

**Table S6: Analysis of microRNAs and mRNAs expression level in 5 subgroups based on 4 genes mutation status**

**Table S7: Expression analysis for DEMs and DEMGs in metastatic colorectal cancer by HCMDB**

**Table S8：Analysis of CIBERSORT scores of 22 types of immune cells in colorectal cancers versus controls**
